# Genetic, Biochemical, and Environmental Factors Associated with Pregnancy Outcomes in Newborns from the Czech Republic

**DOI:** 10.1289/ehp.1002470

**Published:** 2010-10-05

**Authors:** Pavel Rossner, Nana Tabashidze, Miroslav Dostal, Zuzana Novakova, Irena Chvatalova, Milada Spatova, Radim J. Sram

**Affiliations:** Laboratory of Genetic Ecotoxicology, Institute of Experimental Medicine, Academy of Sciences of the Czech Republic, Prague, Czech Republic

**Keywords:** air pollution, biomarkers, genetic polymorphisms, oxidative damage, pregnancy outcomes

## Abstract

**Background:**

Oxidative damage to placental DNA can result in negative pregnancy outcomes, including intrauterine growth restriction (IUGR) and low birth weight (LBW).

**Objective:**

We investigated associations between the levels of 8-oxo-7,8-dihydro-2′-deoxyguanosine (8-oxodG), a marker of oxidative DNA damage, in placental DNA, exposure to air pollutants during pregnancy, genetic polymorphisms in 94 selected genes, and pregnancy outcomes.

**Methods:**

We studied 891 newborns who were IUGR- or LBW-affected or normal weight and were born between 1994 and 1999 in the Czech Republic in two districts with different levels of air pollution.

**Results:**

We found nonsignificantly elevated 8-oxodG levels in the IUGR-affected group compared with the non-IUGR group (*p* = 0.055). Similarly, slightly elevated 8-oxodG levels were found in the LBW-affected group compared with the non-LBW group (*p* < 0.050). In univariate analyses, we identified single nucleotide polymorphisms associated with 8-oxodG levels, IUGR, and LBW. Exposure to particulate matter < 2.5 μm was associated with increased 8-oxodG levels in placental DNA and LBW. However, multivariate-adjusted logistic regression revealed that above-median 8-oxodG levels were the only factor significantly associated with IUGR [OR = 1.56; 95% confidence interval (CI), 1.07–2.37; *p* = 0.022]. Above-median levels of 8-oxodG were associated with LBW (OR = 1.88; 95% CI, 1.15–3.06; *p* = 0.011). Other variables associated with LBW included sex and gestational age of the newborn, maternal smoking, and haplotypes in the promoter region of the gene encoding mannose-binding lectin 2 (*MBL2*). The role of air pollutants in the risk of adverse pregnancy outcomes seemed to be less important.

**Conclusions:**

Levels of 8-oxodG in placental DNA were associated with the risk of IUGR as well as LBW. Newborn’s sex, gestational age, maternal smoking, and genetic polymorphisms in the promoter region of the *MBL2* gene were associated with LBW incidence.

Negative pregnancy outcomes affect millions of newborns worldwide. It is estimated that at least 15% of children are born with low birth weight (LBW): weight < 2,500 g (Gluckman and Hanson 2006). In the Czech population, the incidence of LBW in the 1990s was about 5%, and up to 10% in polluted regions (Sram et al. 1991). According to the recent data, the incidence of LBW increased to 7.11% in 2008 (Institute of Health Information and Statistics of the Czech Republic 2008). A newborn who fails to achieve a weight threshold for his or her gestational age is characterized as small for gestational age (SGA). Intrauterine growth restriction (IUGR) is a syndrome of SGA fetuses who have failed to reach their growth potential for various reasons, including genetic factors, infections, or placental disorders. Therefore, IUGR can be described as a pathological form of SGA (Bamberg and Kalache 2004). LBW and particularly IUGR newborns often suffer from various negative health consequences. Mortality and morbidity have been shown to be increased among newborns whose birth weights are at or below the third percentile of their gestational age (McIntire et al. 1999). Cognitive impairment and the need for special education have also been shown to be increased, as well as a higher incidence of premature birth, among children born small (reviewed by Gluckman and Hanson 2006). Longer-term consequences of LBW include higher risks of cardiovascular diseases, obstructive pulmonary disease, type 2 diabetes mellitus, renal insufficiency, and impaired reproductive functions (Barker 1995; Saenger et al. 2007).

Various factors have been suggested as causes of LBW syndromes. They include medical conditions of the mother, maternal social conditions, fetal problems, abnormalities of the placenta, and environmental factors (Bamberg and Kalache 2004; Saenger et al. 2007). Abnormal pregnancy outcomes may also be associated with specific genetic polymorphisms, dysregulated immune responses, aberrant angiogenesis (Li and Huang 2009), and increased oxidative stress in the placenta and maternal circulation (Poston and Raijmakers 2004).

Among a complex mixture of chemicals present in the ambient air, polycyclic aromatic hydrocarbons (PAHs) are particularly important, because some of them are carcinogenic (c-PAHs) and may be directly responsible for the increased incidence of cancer due to PAH–DNA adduct formation and subsequent mutation induction (Lewtas 2007). PAHs, as well as other chemicals in the air, are adsorbed onto the surface of particulate matter (PM) of various aerodynamic diameters. Exposure to PM < 2.5 μm (PM_2.5_) is associated with increased all-cause lung cancer and cardiopulmonary mortality (Pope et al. 2002). A major consequence of PM exposure in humans is the generation of reactive oxygen species (ROS), which can occur via various mechanisms that include metabolism, redox cycling, and inflammation and result in oxidative damage to DNA and other macromolecules (Moller et al. 2008).

Oxidative stress mediated by PM has been proposed as one of the mechanisms responsible for adverse pregnancy outcomes (Kannan et al. 2006). Increased oxidative stress, accompanied by reduced translation of proteins, including key signaling molecules, is present in the placentas of IUGR pregnancies (Yung et al. 2008). Oxidative stress causes injury to chorionic villi, reducing the functional mass of the syncytiotrophoblast and limiting the capacity of the villi to mediate nutrient transport, thus contributing to IUGR induction (Scifres and Nelson 2009). Inflammation, also related to oxidative stress induction, is another key process associated with LBW syndromes. Whereas normal pregnancy is a state of permanent mild inflammation, in adverse pregnancy outcomes, the inflammation is increased above normal levels (Li and Huang 2009). The resulting inflammatory state is accompanied by maternal leukocyte activation, the release of cytokines from immune cells and uteroplacental tissues, and endothelial cell activation, as well as immune–coagulation interactions. Activated immune cells express various molecules that are important in the formation of a dysfunctional placenta, resulting in excessive trophoblast apoptosis, shallow trophoblast invasion, and impaired spiral artery remodeling (Li and Huang 2009). These processes cause adverse pregnancy outcomes.

The aim of our study was to investigate factors associated with LBW and IUGR risks in a group of 891 newborns born in two districts of the Czech Republic with different levels of air pollution. The analyzed factors included the levels of air pollutants [benzo[*a*]pyrene (B[*a*]P), PM_2.5_] during pregnancy, 8-oxo-7,8-dihydro-2′-deoxyguanosine (8-oxodG) levels in placental DNA, and a panel of 576 single nucleotide polymorphisms (SNPs) in 97 genes related to DNA repair, oxidative stress, xenobiotic metabolism, and immune functions. We hypothesized that air pollution induces oxidative DNA damage, which, in turn, increases the risk of adverse pregnancy outcomes. These effects may be modulated by SNPs in selected genes. Our principal aim was to determine whether oxidative stress was a major factor affecting LBW and IUGR in our cohort.

## Materials and Methods

### Subjects and samples

For our study, 1,200 subjects were randomly selected from a large case–control study conducted between 1994 and 1999 in two districts in the Czech Republic (Teplice and Prachatice) that have different levels of air pollution (Dejmek et al. 1999, 2000). The Teplice district is located in Northern Bohemia, and at the time of the study it was one of the most polluted regions of the Czech Republic. Air pollution arose mostly from chemical industry and brown coal power plants. The District of Prachatice is a rural region in Southern Bohemia with little industry and low levels of air pollution [Supplemental Material, Tables 2 and 3 (doi:10.1289/ehp.1002470)]. The aim of the original study was to evaluate the effect of ambient PAHs and fine particles on pregnancy outcome. The criteria for the selection of samples for this study were that the proportion of both IUGR- and LBW-affected newborns would be 10–15%. After removing the samples for which all relevant information was not available, the final cohort consisted of 891 subjects, among them 15.8% newborns with IUGR and 14.5% newborns with LBW.

All mothers included in the study signed informed consent forms and could cancel their participation at any time, according to the Helsinki II declaration. The ethical committee of the Institute of Experimental Medicine approved the study. The subjects analyzed in our study were single births that occurred in each district between April 1994 and March 1999. Preterm births (< 37 weeks gestation) were not included. The subjects were of two ethnic origins: European (749 newborns) or Romani (142 newborns). Detailed health, personal, and lifestyle information was obtained from self-administered maternal questionnaires and from medical records. The Romani ethnicity was determined according to the answer in the maternal questionnaires. An IUGR birth was defined as one in which the newborn had a birth weight below the 10th percentile, by sex and gestational age, for live births in the Czech Republic (Dejmek et al. 1999). The gestational age was estimated by gynecologists using each woman’s information on her last menstrual period, prenatal visits, and ultrasound examinations (Dejmek et al. 1999). LBW newborns had a birth weight < 2,500 g. Placentas were collected at the time of birth. The villus parenchyma sections were obtained by dissecting a 1.5-cm cubic-shaped segment (approximately 5 cm away from the site of cord insertion) and then splitting it into three equal parts: maternal (including thin basal plate), middle, and fetal (including the chorionic plate). The middle sections (one sample per placenta) were frozen and stored at −80°C until further processing.

### Air pollution monitoring

Concentrations of ambient air pollutants in both districts were measured using a Versatile Air Pollution Sampler (VAPS) (Pinto et al. 1998). The VAPS continuously monitored the levels of c-PAHs and PM_2.5_. PAHs were extracted from filters, and a quantitative chemical analysis of c-PAHs, including benz[*a*]anthracene, chrysene, benzo[*b*]fluoranthene, benzo[*k*]fluoranthene, B[*a*]P, dibenzo[*a,h*]anthracene, benzo[*g,h,i*]perylene, and indeno[*1,2,3-cd*]pyrene was performed by high performance liquid chromatography with fluorescence detection according to the U.S. Environmental Protection Agency method (U.S. Environmental Protection Agency 1999).

### DNA isolation and analysis of 8-oxodG adducts

DNA was isolated from the placenta essentially as described previously (Rossner et al. 2009). Vacuum-dried DNA pellets were stored at −80°C until further processing. Oxidative DNA damage, measured as levels of 8-oxodG in placental DNA, was analyzed using competitive ELISA with the primary antibody N45.1 (concentration 0.2 μg/mL; JaICA, Haruoka, Japan) as described (Rossner et al. 2009). Each sample was analyzed in triplicate. 8-OxodG levels were expressed as the number of 8-oxodG molecules per 10^5^ guanosine molecules (8-oxodG/10^5^ dG).

### Analysis of genetic polymorphisms

DNA samples for the analysis of genetic polymorphisms were dissolved in TE buffer (10 mM Tris base, 0.1 mM EDTA, pH 8.0) and diluted to a final concentration of 50 ng/μL. The samples were stored at −80°C until analysis. Genetic polymorphisms were assessed in 97 genes.

The presence/absence of the glutathione *S*-transferase M1 (*GSTM1*) and *GSTT1* genes was assessed by the TaqMan Real-Time PCR Assay [TaqMan Gene Copy Number Assays (PN4331182); Applied Biosystems, Carlsbad, CA, USA]. Although the method allows for the analysis of the exact number of the respective genes in the sample, for the purposes of our study we split our samples into two groups: *GSTM1/GSTT1*–null and *GSTM1/GSTT1*–positive samples (this group contained all samples having at least one copy of the gene). The assay was performed on a 7900HT system (Applied Biosystems) according to the manufacturer’s recommendation.

SNPs in the remaining 95 genes were analyzed by the GoldenGate genotyping assay developed by Illumina (San Diego, CA, USA) on custom-made 96-sample panels. SNPs that could be potentially associated with adverse pregnancy outcomes, oxidative stress, or the response of the organism to air pollutants were selected to be analyzed. They included genes related to the protection of the organism against oxidative damage, genes responsible for the metabolism of xenobiotics, DNA repair genes, and genes affecting immune and inflammatory responses. SNPs in these genes were selected from the SNP500Cancer Database (http://snp500cancer.nci.nih.gov/). The criterion for the selection of an SNP was that the frequency of the minor allele should not be < 5%. We selected a total of 768 SNPs for a custom-made panel. We performed the GoldenGate genotyping assay on a BeadStation 500GX system (Illumina) according to the manufacturer’s instructions and analyzed the results using the BeadStudio software. We first excluded samples and SNPs that were not successfully analyzed based on the following parameters: p10 GC, all samples with a score < 0.40 were removed; Call Frequency, all SNPs with a score < 0.60 were excluded from further analysis. The SNPs and samples that remained were checked manually for proper clustering, and the final results were exported to an Excel file. After analyzing the results, we removed 138 DNA samples for which we were not able to determine the genetic polymorphism and 40 SNPs that did not cluster properly.

### Statistical analysis

Although we started our study with 1,200 samples, statistical analysis was performed on 891 samples for which all relevant data were available. We first compared 8-oxodG levels between districts for both ethnicities and for pregnancy outcomes using the Mann–Whitney *U* test. We checked the distribution of alleles for individual SNPs for Hardy–Weinberg equilibrium using a chi-square test and removed an additional 153 SNPs that were not in equilibrium. The final number of genetic polymorphisms used for the statistical analysis was 577 in 94 genes [listed in Supplemental Material, Table 1 (doi:10.1289/ehp.1002470)]. The variables that were not distributed normally were log transformed. Using binary linear or logistic regression, we analyzed the association of *a*) genetic polymorphisms with 8-oxodG levels, *b*) genetic polymorphisms with pregnancy outcomes, *c*) exposure to environmental pollutants (B[*a*]P, PM_2.5_) with 8-oxodG levels, *d*) exposure to air pollution with pregnancy outcomes, and *e*) lifestyle, socioeconomic, and other factors [newborn’s ethnicity, sex, district, length of gestation; mother’s age, body mass index (BMI), smoking during pregnancy, and length of education; father’s smoking and length of education] with pregnancy outcomes. Finally, we used the above-mentioned variables that were significantly associated with 8-oxodG and pregnancy outcomes in a multivariate logistic regression analysis. For logistic regression estimates, we transformed continuous variables into a two-level scale using medians.

To correct for multiple comparisons, we used the FDR method (QVALUE software) (Storey and Tibshirani 2003). Haplotypes in the promoter region of the mannose-binding lectin 2 (*MBL2*) gene were estimated using PHASE 2.1 software (Stephens and Scheet 2005; Stephens et al. 2001). All other analyses were performed using SPSS 17.0 software (SPSS Inc., Chicago, IL, USA).

## Results

The aim of our study was to test the hypothesis that exposure to air pollution during pregnancy induces 8-oxodG levels, which are associated with adverse pregnancy outcomes (IUGR, LBW). Pregnancy outcomes and 8-oxodG levels are further affected by genetic polymorphisms, lifestyle, and socioeconomic and other factors. These relationships are depicted in Supplemental Material, Figure 1 (doi:10.1289/ehp.1002470). As illustrated in Supplemental Material, Figure 2, another possibility is that 8-oxodG formation is not an intermediate step in the air pollution-adverse pregnancy outcome pathway, but rather acts as an independent factor contributing to IUGR and/or LBW.

The distribution of lifestyle, socioeconomic, and other characteristics of the subjects enrolled in the study with regard to adverse pregnancy outcomes is shown in Table 1. The proportion of IUGR- and LBW-affected newborns was higher in the Romani than in the European population (Romani: 39 IUGR vs. 103 non-IUGR newborns; 46 LBW vs. 96 non-LBW newborns; European: 102 IUGR vs. 647 non-IUGR newborns; 83 LBW vs. 666 non-LBW newborns). Mothers who smoked during pregnancy tended to have IUGR-and/or LBW-affected newborns (smokers: 81 IUGR vs. 326 non-IUGR newborns; 81 LBW vs. 326 non-LBW newborns; nonsmokers: 59 IUGR vs. 423 non-IUGR newborns; 47 LBW vs. 435 non-LBW newborns). Being a single mother was associated with a higher proportion of IUGR- and LBW-affected newborns (single: 30 IUGR vs. 111 non-IUGR; 36 LBW vs. 105 non-LBW; married: 111 IUGR vs. 634 non-IUGR; 92 LBW vs. 653 non-LBW) (Table 1). Of 891 subjects, 674 pregnancies were normal, 88 newborns were IUGR- but not LBW-affected, 76 newborns were born with LBW only, and 53 subjects were affected by both IUGR and LBW (data not shown). The basic characteristics of the analyzed samples with regard to 8-oxodG levels are presented in Table 2. We did not find any difference in 8-oxodG between the districts, despite higher levels of air pollutants in the Teplice region, which may induce oxidative DNA damage. In addition, 8-oxodG levels did not differ between the two ethnic groups. In placental samples from IUGR subjects, we observed an increase in 8-oxodG levels that was on the borderline of significance (median 8-oxodG/10^5^ dG = 2.03, range, 0.29–6.17; median 8-oxodG/10^5^ dG = 1.74, range, 0.20–6.50 for IUGR and non-IUGR samples, respectively; *p* = 0.055). LBW was associated with significantly higher oxidative DNA damage (median 8-oxodG levels/10^5^ dG = 2.25, range, 0.27–6.28; median 8-oxodG levels/10^5^ dG = 1.75, range, 0.20–6.50 for LBW and non-LBW samples, respectively; *p* < 0.05).

We compared exposure to environmental pollutants during the individual months of pregnancy for IUGR and non-IUGR as well as LBW and non-LBW subjects. Although no significant differences were observed for IUGR and non-IUGR newborns exposed to both B[*a*]P and PM_2.5_ (data not shown) or for LBW and non-LBW children exposed to B[*a*]P (data not shown), LBW-affected children were exposed to higher concentrations of PM_2.5_ during the first to fifth months of pregnancy (Table 3). The concentrations of B[*a*]P during the individual months of pregnancy for all subjects and separately for mothers living in the Prachatice and Teplice districts are shown in Supplemental Material, Table 2 (doi:10.1289/ehp.1002470). The corresponding data for PM_2.5_ are reported in Supplemental Material, Table 3. The levels of air pollutants were significantly higher in the Teplice district for all periods, with the exception of the B[*a*]P levels in the third month of pregnancy.

To test our hypothesis, we first performed univariate analyses of selected variables (e.g., SNPs, oxidative stress, air pollution, lifestyle, socioeconomic and other factors, and pregnancy outcomes). The list of genes and SNPs significantly associated with LBW after correction for multiple comparisons is shown in Supplemental Material, Table 4 (doi:10.1289/ehp.1002470). We found four genes that modified the incidence of LBW: the gene encoding *MBL2*, chemokine (C-C) motif ligand 7 (*CCL7*), C-C motif ligand 11 (*CCL11*), and C-C motif ligand 18 (*CCL18*). For the *MBL2* gene, we detected 10 SNPs associated with an increased risk of LBW. These SNPs, located in the promoter region of the *MBL2* gene, formed three haplotypes with frequencies of 77.7% (haplotype ATAATCCGCT), 22.1% (haplotype GCGGAATAGA), and 0.2% (haplotype ACGGAATAGA). The homozygous combination ATAATCCGCT/ATAATCCGCT found in 59.1% of subjects was prevalent. Other combinations of haplotypes were detected in 40.9% of placental DNA samples. Table 4 summarizes the estimated effect of haplotypes in the promoter region of *MBL2* on the LBW risk. Subjects not carrying the prevailing homozygous combination of the ATAATCCGCT haplotype were at significantly higher risk of LBW. We also identified genes and SNPs significantly associated with IUGR as well as 8-oxodG levels. However, after FDR correction, these associations were no longer evident (data not shown).

Using univariate analysis, we further investigated the effect of exposure to B[*a*]P and PM_2.5_ during pregnancy on 8-oxodG levels. 8-OxodG levels in placental DNA was not affected by B[*a*]P (data not shown), whereas PM_2.5_ exposure during the second month of pregnancy was significantly associated with 8-oxodG levels in placental DNA [odds ratio (OR) = 1.68; 95% confidence interval (CI), 1.28–2.19; *p* < 0.001].

In univariate analyses, 8-oxodG levels in placental DNA were significantly associated with IUGR (OR = 1.47; 95% CI, 1.02–2.12; *p* = 0.037; Table 5), but not with LBW (Table 6). Other factors associated with IUGR included ethnicity, maternal BMI, maternal smoking, and the mother’s and father’s length of education (Table 5). Exposure to PM_2.5_ was not associated with IUGR (data not shown). District, ethnicity, sex and gestational age of the child, maternal BMI, smoking, marital status, and vitamin intake during pregnancy, paternal length of education and smoking, and exposure to PM_2.5_ in the first month of pregnancy were among variables associated with the risk of LBW (Table 6). B[*a*]P exposure affected neither IUGR nor LBW (data not shown).

The findings from multivariate-adjusted logistic regression investigating the factors associated with IUGR are reported in Table 5. The results indicate that 8-oxodG levels remained the only variable significantly associated with the risk of IUGR (OR = 1.57; 95% CI, 1.06–2.34; *p* = 0.026). Factors associated with LBW after multivariate adjustment include 8-oxodG levels (OR = 1.83; 95% CI, 1.12–3.00; *p* = 0.017), sex (OR = 1.96; 95% CI, 1.21–3.18; *p* = 0.006), gestational age (OR = 0.05; 95% CI, 0.03–0.11; *p* < 0.001), maternal smoking (OR = 0.52; 95% CI, 0.29–0.91; *p* = 0.023), and the haplotype in the *MBL2* gene (OR = 2.59; 95% CI, 1.59–4.20; *p* < 0.001) (Table 6).

## Discussion

The aim of our study was to investigate the role of oxidative DNA damage, possibly induced by exposure to air pollution (measured as concentrations of B[*a*]P and PM_2.5_) and modulated by genetic polymorphisms in selected relevant genes, in the risk of adverse pregnancy outcomes. Our results indicate that increased 8-oxodG levels in placental DNA are an independent factor associated with the risk of IUGR as well as LBW. The role of air pollution in our samples seems to be minor.

Oxidative stress during pregnancy is a normal physiological condition, and the fetus possesses mechanisms that help to minimize the deleterious effects of the production of ROS. These mechanisms include the expression of the antioxidant enzymes catalase, glutathione peroxidase, GST, thiol/disulfide oxidoreductase, and superoxide dismutase, as well as the accumulation of antioxidants (glutathione and vitamins C and E) (Myatt and Cui 2004). However, in the case of excessive ROS production, apoptosis in the trophoblast is induced (Smith et al. 1999), which in turn increases the risk of IUGR (Myatt and Cui 2004). Several authors have reported increased levels of various oxidative stress markers during pregnancy and subsequent unfavorable pregnancy outcomes. The levels of malondialdehyde and xanthine oxidase were elevated in maternal and umbilical cord plasma and placental tissues with IUGR when compared with controls (Biri et al. 2007). Elevated urinary 8-oxodG levels during early pregnancy were predictors of LBW (Peter Stein et al. 2008; Potdar et al. 2009). Other authors reported associations of increased levels of oxidative stress markers measured in the third trimester of pregnancy with unfavorable pregnancy outcomes (Orhan et al. 2003; Scholl and Stein 2001). In our study, we measured 8-oxodG levels in placental DNA at delivery, with similar results of elevated oxidative DNA damage in both IUGR and LBW newborns.

Apart from endogenous factors, oxidative stress may be also affected by various exogenous stimuli, including ambient air pollution. Numerous studies have shown that exposure to air pollutants increases the levels of oxidative stress markers (Avogbe et al. 2005; reviewed by Kelly 2003; Loft et al. 2008; Rossner et al. 2007, 2008a, 2008b; Sorensen et al. 2003a, 2003b). Pollutants are mostly adsorbed onto PM of various sizes. After entering the organism, PM exerts its negative effect either by means of chemical compounds adsorbed to PM or via inflammation-related pathways directly linked with ROS production and thus oxidative stress induction (Risom et al. 2005). In agreement with these observations, we found that 8-oxodG levels were associated with exposure to PM_2.5_ during the second month of pregnancy; this association, however, vanished in the final multivariate-adjusted model. No significant associations were detected for B[*a*]P exposure.

The effect of various ambient air pollutants on adverse pregnancy outcomes has been analyzed in numerous studies with conflicting results. Exposure to PM < 10 μm during the first month of pregnancy was associated with increased risk of IUGR (Dejmek et al. 1999). Similarly, c-PAH exposure during the first gestational month significantly elevated IUGR (Dejmek et al. 2000). LBW and the prevalence of premature births were associated with the concentrations of sulfur dioxide and total suspended particles during individual trimesters of pregnancy: LBW was associated with concentrations of both sulfur dioxide and total suspended particles in all three trimesters, whereas prematurity was associated with sulfur dioxide in three trimesters and with total suspended particles only in the first trimester of pregnancy (Bobak 2000). LBW was increased in association with residence within 50 m of highways, but no direct effect of PM_2.5_ on LBW was found (Brauer et al. 2008). The results from other studies are also discussed in reviews (Maisonet et al. 2004; Sram et al. 2005). In this study, we observed evidence of an effect of air pollution on pregnancy outcomes, specifically the association of PM_2.5_ concentrations in the first month of pregnancy and LBW. The estimated effect of PM_2.5_ and B[*a*]P on IUGR was not significant. We did not observe any influence of B[*a*]P exposure on LBW. However, a significant association between exposure to PM_2.5_ and LBW did not remain after adjusting for other variables possibly affecting adverse pregnancy outcomes. Because the samples analyzed in this study partially overlap with the samples described by Dejmek et al. (1999, 2000), it would be reasonable to expect similar results for the association between exposures to PM_2.5_ and B[*a*]P and pregnancy outcomes. We do not have any definite explanation for the conflicting results we obtained. We speculate that the reduced sample size in our study when compared with the original project may be one of the reasons. In addition, the samples in studies by Dejmek et al. were collected earlier in the 1990s, when the concentrations of air pollutants, particularly in the Teplice region, were higher than at the end of the decade. Finally, Dejmek et al. split the concentrations of PM_2.5_ into tertiles. IUGR was associated only with the highest concentrations of PM_2.5_ (the third tertile; Dejmek et al. 1999). In contrast, we used the median in our calculations and therefore did not test separately the effect of PM_2.5_ levels in the third tertile on the incidence of IUGR or LBW.

Genetic polymorphisms may modulate the response of the organism to various factors and affect its ability to cope with adverse conditions. Given the sample size that we analyzed, we were able to use a moderately high-throughput method of SNP detection that allowed us to assess 574 SNPs in 95 genes and 2 gene deletions.

Although we did not detect any SNP associated with either oxidative DNA damage or IUGR, in univariate analysis we found four genes (*MBL2*, *CCL7*, *CCL11*, and *CCL18*) modifying the incidence of LBW. After adjusting for other variables, only *MBL2* remained a significant factor affecting LBW. The *MBL2* gene encodes MBL, a protein that plays an important role in innate immunity. The protein has diverse functions that include complement activation, opsonophagocytosis, the modulation of inflammation, and the clearance of apoptotic cells (Dommett et al. 2006). It provides the first-line defense against microorganisms. Polymorphisms in exon 1 that affect the levels of MBL in serum have been described (Garred 2008). The *MBL2* promoter 1 has been shown to be highly polymorphic. In particular, the variant alleles in three SNPs (rs11003125, rs7096206, and rs7095891) cause lower activity of the promoter (Garred 2008), thus decreasing the concentrations of MBL in serum. These SNPs were associated with shorter gestational age (van de Geijn et al. 2008) as well as with LBW (Thevenon et al. 2009). In our study, we identified 10 SNPs in the promoter region of *MBL2* that were associated with LBW. These SNPs form three haplotypes. The homozygous combination of the most frequent haplotype was inversely associated with LBW. Although the SNPs affecting LBW differed from those identified previously (Thevenon et al. 2009), they are also in the promoter region, and thus our results support the data of Thevenon et al. (2009). These authors also analyzed MBL concentrations in serum and found that lower levels are associated with polymorphisms that increased the LBW risk. This is in contrast with van de Geijn et al. (2008), who reported that shorter gestational age was associated with higher serum levels of MBL. Because we did not measure serum levels of MBL, we can only speculate that lower frequency haplotypes modify the activity of the promoter, thus changing the serum levels of MBL. This may negatively affect immune responses or induce inflammation, ultimately resulting in LBW.

Whereas univariate analyses indicate that only IUGR is associated with 8-oxodG levels in placental DNA, multivariate-adjusted analyses identified both IUGR and LBW as adverse pregnancy outcomes where oxidative DNA damage appears to play a significant role. No other variable significantly affecting IUGR was found. Thus, it seems that oxidative DNA damage resulting from various sources is a key event leading to the IUGR syndrome. On the other hand, LBW was affected by several variables. We saw the strongest association with gestational age, sex, and the haplotype in the promoter region of *MBL2*. Based on these findings, we cannot confirm our hypothesis that 8-oxodG is an intermediate in the air pollution-adverse pregnancy outcome pathway [Supplemental Material, Figure 1 (doi:10.1289/ehp.1002470)]. It seems rather more probable that oxidative damage is one of the independent factors (a key factor in the case of IUGR) that collectively contribute to adverse pregnancy outcomes.

## Conclusions

Our results suggest that levels of 8-oxodG in placental DNA play a significant role in both IUGR- and LBW-affected pregnancies. IUGR is associated mostly with 8-oxodG, whereas LBW is further affected by the sex and gestational age of the child, maternal smoking, and genetic polymorphisms in the *MBL2* gene, the protein product of which is an important factor in innate immunity. The role of air pollution in the risk for both adverse pregnancy outcomes in this particular data set seems to be less important.

## Figures and Tables

**Table 1:** Lifestyle and socioeconomic characteristics of the study group.

	IUGR		Non-IUGR		LBW		Non-LBW	
	*n* (%)	Mean ± SD	*n* (%)	Mean ± SD	*n* (%)	Mean ± SD	*n* (%)	Mean ± SD
Characteristics of mother and child								

District								
Prachatice	42 (4.7)		255 (28.6)		19 (2.1)		278 (31.2)	
Teplice	99 (11.1)		495 (55.6)		110 (12.3)		484 (54.4)	
Ethnicity								
European	102 (11.4)		647 (72.6)		83 (9.3)		666 (74.7)	
Romani	39 (4.4)		103 (11.6)		46 (5.2)		96 (10.8)	
Child’s sex								
Male	68 (7.6)		386 (43.3)		55 (6.2)		399 (44.8)	
Female	73 (8.2)		364 (40.9)		74 (8.3)		363 (40.7)	
Gestational age (weeks)		39.1 ± 1.78		38.9 ± 2.28		35.8 ± 3.03		39.5 ± 1.47
Mother’s age at delivery (years)		24.2 ± 5.04		25.0 ± 4.79		24.7 ± 5.43		24.9 ± 4.73
Mother’s BMI (kg/m^2^)		21.4 ± 3.74		22.5 ± 3.73		21.4 ± 3.40		22.4 ± 3.79
Mother’s smoking								
Yes	81 (9.1)		326 (36.7)		81 (9.1)		326 (36.7)	
No	59 (6.6)		423 (47.6)		47 (5.3)		435 (48.9)	
Length of mother’s education								
≤ 11 years	102 (11.5)		442 (50.0)		84 (9.5)		460 (52.1)	
> 11 years	36 (4.1)		304 (34.4)		41 (4.6)		299 (33.8)	
Marital status								
Single	30 (3.4)		111 (12.5)		36 (4.1)		105 (11.9)	
Married	111 (12.5)		634 (71.6)		92 (10.3)		653 (73.7)	
Vitamin intake during pregnancy								
≤ 2 times per week	82 (9.2)		380 (42.7)		84 (9.4)		378 (42.4)	
> 2 times per week	59 (6.6)		370 (41.5)		45 (5.1)		384 (43.1)	

Characteristics of father								

Length of father’s education								
≤ 11 years	97 (11.4)		440 (51.6)		88 (10.3)		449 (52.7)	
> 11 years	33 (3.9)		282 (33.1)		29 (3.4)		286 (33.6)	
Father’s smoking								
Yes	87 (10.1)		414 (48.0)		81 (9.4)		420 (48.7)	
No	47 (5.5)		314 (36.4)		36 (4.2)		325 (37.7)	

**Table 2:** 8-OxodG levels in placental DNA of all analyzed samples and in subgroups by district, ethnicity, and pregnancy outcome.

		8-OxodG/10^5^ dG		
Variable	*n*	Mean ± SD	Median (range)	*p*-Value^a^
All subjects	891	2.11 ± 1.31	1.80 (0.20–6.50)	
District				
Prachatice	297	2.06 ± 1.33	1.76 (0.21–6.50)	0.280
Teplice	594	2.14 ± 1.30	1.81 (0.20–6.43)	
Ethnicity				
European	749	2.09 ± 1.31	1.80 (0.20–6.50)	0.272
Romani	142	2.23 ± 1.35	1.81 (0.27–6.13)	
Pregnancy outcome: IUGR				
Non-IUGR	750	2.08 ± 1.30	1.74 (0.20–6.50)	0.055
IUGR	141	2.29 ± 1.34	2.03 (0.29–6.17)	
Pregnancy outcome: LBW				
Non-LBW	762	2.07 ± 1.28	1.75 (0.20–6.50)	< 0.05
LBW	129	2.37 ± 1.46	2.25 (0.27–6.28)	

a For comparison of medians by the Mann–Whitney *U* test.

**Table 3:** Concentrations of PM_2.5_ during pregnancy for non-LBW and LBW subjects.

	Concentration of PM_2.5_ (μg/m^3^)				
	Non-LBW		LBW		
Pregnancy month	Mean ± SD	Median (min, max)	Mean ± SD	Median (min, max)	*p*-Value^a^
1	28.8 ± 12.8	26.2 (1.13, 84.6)	33.2 ± 13.1	30.2 (11.0, 72.6)	0.000
2	29.2 ± 12.6	26.5 (1.09, 82.4)	33.6 ± 12.6	32.1 (12.4, 72.6)	0.000
3	30.2 ± 13.6	27.2 (1.09, 84.6)	32.6 ± 13.6	30.2 (8.02, 83.6)	0.035
4	29.3 ± 13.7	26.0 (1.13, 86.1)	31.3 ± 11.4	30.0 (10.6, 64.6)	0.021
5	29.2 ± 12.7	26.8 (1.13, 84.5)	33.4 ± 14.8	31.0 (11.0, 82.4)	0.004
6	29.5 ± 13.8	26.4 (3.25, 86.1)	30.4 ± 12.3	27.0 (10.6, 84.6)	0.148
7	26.2 ± 13.6	26.2 (3.25, 84.8)	29.7 ± 13.3	25.9 (4.48, 76.3)	0.606
8	30.3 ± 16.0	25.8 (3.83, 86.1)	30.6 ± 13.7	26.2 (9.94, 80.5)	0.389
9	28.5 ± 14.0	25.1 (1.09, 84.8)	29.6 ± 13.4	26.2 (5.99, 73.8)	0.183

Abbreviations: max, maximum; min, minimum.

a Comparison of PM_2.5_ concentrations between non-LBW and LBW subjects using the Mann–Whitney *U* test.

**Table 4:** Results from univariate logistic regression of haplotypes in the promoter region of *MBL2* modifying the incidence of LBW.

Haplotype	Non-LBW (*n*)	LBW (*n*)	Crude OR (95% CI)	*p-*Value^a^
ATAATCCGCT/ATAATCCGCT	471	56	Reference	
Other combinations	291	73	2.11 (1.45–3.08)	< 0.001
ATAATCCGCT/GCGGAATAGA				
GCGGAATAGA/GCGGAATAGA				
ATAATCCGCT/ACGGAATAGA				

a *p*-Value estimated using logistic regression analysis.

**Table 5:** Results from univariate and multivariate-adjusted logistic regression of factors associated with IUGR.

	Univariate analysis		Multivariate analysis	
Variable	Crude OR (95% CI)	*p-*Value^a^	OR^b^ (95% CI)	*p-*Value^a^
8-OxodG/10^5^ dG (below/above median)	1.47 (1.02–2.12)	0.037	1.57 (1.06–2.34)	0.026
District (Prachatice/Teplice)	1.21 (0.82–1.80)	0.331	0.90 (0.57–1.44)	0.669
Ethnicity (European/Romani)	2.43 (1.59–3.72)	< 0.001	1.51 (0.85–2.68)	0.157
Sex (male/female)	1.14 (0.79–1.63)	0.480	1.12 (0.76–1.66)	0.557
Gestational age (below/above median)	0.96 (0.67–1.39)	0.842	1.13 (0.74–1.71)	0.580
Mother’s age at delivery (below/above median)	0.71 (0.50–1.02)	0.067	0.94 (0.64–1.47)	0.891
Mother’s BMI (below/above median)	0.66 (0.46–0.95)	0.025	0.74 (0.50–1.10)	0.137
Mother’s smoking (no/yes)	1.78 (1.24–2.57)	0.002	1.31 (0.84–2.05)	0.234
Mother’s education [years (below/above median)]	0.51 (0.34–0.77)	0.001	0.62 (0.37–1.02)	0.060
Marital status (single/married)	0.65 (0.41–1.02)	0.059	1.12 (0.62–2.02)	0.711
Vitamin intake during pregnancy (times per week; below/above median)	0.74 (0.51–1.06)	0.103	0.96 (0.64–1.45)	0.847
Father’s education [years (below/above median)]	0.53 (0.35– 0.81)	0.003	0.78 (0.47–1.29)	0.336
Father’s smoking (no/yes)	1.40 (0.96–2.06)	0.083	1.05 (0.66–1.66)	0.852

a *p*-Value estimated using logistic regression analysis.

b Adjusted for all the factors in the table.

**Table 6:** Results from univariate and multivariate-adjusted logistic regression of factors associated with LBW.

	Univariate analysis		Multivariate analysis	
Variable	Crude OR (95% CI)	*p-*Value^a^	OR^b^ (95% CI)	*p-*Value^a^
8-OxodG/10^5^ dG (below/above median)	1.36 (0.93–1.98)	0.109	1.83 (1.12–3.00)	0.017
District (Prachatice/Teplice)	3.33 (2.00–5.53)	< 0.001	1.77 (0.90–3.45)	0.097
Ethnicity (European/Romani)	3.80 (2.50–5.79)	< 0.001	0.86 (0.42–1.74)	0.668
Sex (male/female)	1.48 (1.02–2.16)	0.042	1.96 (1.21–3.18)	0.006
Gestational age (below/above median)	0.05 (0.03–0.10)	< 0.001	0.05 (0.03–0.11)	< 0.001
Mother’s age at delivery (below/above median)	1.07 (0.74–1.56)	0.721	1.39 (0.83–2.34)	0.214
Mother’s BMI (below/above median)	0.61 (0.42–0.89)	0.011	0.70 (0.42–1.14)	0.149
Mother’s smoking (no/yes)	2.30 (1.56–3.39)	< 0.001	1.94 (1.10–3.44)	0.023
Mother’s education [years (below/above median)]	0.75 (0.50–1.12)	0.161	1.46 (0.79–2.69)	0.228
Marital status (single/married)	0.41 (0.27–0.64)	< 0.001	0.70 (0.36–1.38)	0.304
Vitamin intake during pregnancy [times per week (below/above median)]	0.53 (0.36–0.78)	0.001	0.68 (0.41–1.13)	0.134
Father’s education [years (below/above median)]	0.52 (0.33–0.81)	0.004	0.78 (0.41–1.46)	0.434
Father’s smoking (no/yes)	1.74 (1.15–2.65)	0.009	0.77 (0.42–1.39)	0.384
PM_2.5_ levels [first month of pregnancy (below/above median)]	1.90 (1.29–2.81)	0.001	0.89 (0.52–1.54)	0.685
rs8081047 (CC vs. CT + TT)	2.40 (1.54–3.74)	< 0.001	1.46 (0.11–18.6)	0.773
rs16969415 (TT vs. CT + CC)	2.41 (1.54–3.79)	< 0.001	0.78 (0.06–10.5)	0.854
rs854477 (GG vs. AG + AA)	0.48 (0.33–0.71)	< 0.001	1.31 (0.80–2.15)	0.289
Haplotype in *MBL2* (ATAATCCGCT/ATAATCCGCT vs. other combinations, Table 4)	2.11 (1.45–3.08)	< 0.001	2.59 (1.59–4.20)	< 0.001

a *p*-Value estimated using logistic regression analysis.

b Adjusted for all the factors in the table.
